# Simultaneous species detection and discovery with environmental DNA metabarcoding: A freshwater mollusk case study

**DOI:** 10.1002/ece3.11020

**Published:** 2024-02-16

**Authors:** Daniel D. Vanderpool, Taylor M. Wilcox, Michael K. Young, Kristine L. Pilgrim, Michael K. Schwartz

**Affiliations:** ^1^ USDA Forest Service Rocky Mountain Research Station, National Genomics Center for Wildlife and Fish Conservation Missoula Montana USA

**Keywords:** environmental DNA species detection, metabarcoding, springsnails

## Abstract

Environmental DNA (eDNA) sampling is a powerful tool for rapidly characterizing biodiversity patterns for specious, cryptic taxa with incomplete taxonomies. One such group that are also of high conservation concern are North American freshwater gastropods. In particular, springsnails of the genus *Pyrgulopsis* (Family: Hydrobiidae) are prevalent throughout the western United States where >140 species have been described. Many of the described species are narrow endemics known from a single spring or locality, and it is believed that there are likely many additional species which have yet to be described. The distribution of these species across the landscape is of interest because habitat loss and degradation, climate change, groundwater mining, and pollution have resulted in springsnail imperilment rates as high as 92%. Determining distributions with conventional sampling methods is limited by the fact that these snails are often <5 mm in length with few distinguishing morphological characters, making them both difficult to detect and to identify. We developed an eDNA metabarcoding protocol that is both inexpensive and capable of rapid, accurate detection of all known *Pyrgulopsis* species. When compared with conventional collection techniques, our pipeline consistently resulted in detection at sites previously known to contain *Pyrgulopsis* springsnails and at a cost per site that is likely to be substantially less than the conventional sampling and individual barcoding that has been done historically. Additionally, because our method uses eDNA extracted from filtered water, it is non‐destructive and suitable for the detection of endangered species where “no take” restrictions may be in effect. This effort represents both a tool which is immediately applicable to taxa of high conservation concern across western North America and a case study in the broader application of eDNA sampling for landscape assessments of cryptic taxa of conservation concern.

## Graphical abstract

Springsnails of the genus *Pyrgulopsis* (Family: Hydrobiidae) are prevalent throughout the western United States where >140 species have been described. Many of the described species are narrow endemics known from a single spring or locality, and it is believed that there are likely many additional species which have yet to be described. We developed a metabarcoding strategy that uses modified COI primers for sensitive detection of *Pyrgulopsis* sp. springsnails that can be rapidly deployed at landscape scales for use by land managers and conservation efforts.

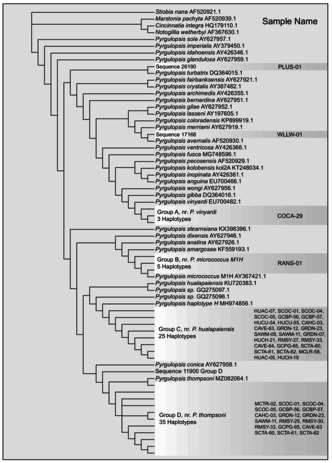

## 1 INTRODUCTION

Environmental DNA sampling—the inferences of species presence from genetic material in the environment—is unique among sampling methods in that there is the opportunity to characterize genetic diversity within entire ecological communities, capturing information about described and undescribed taxa (Deiner et al., 2017). We can simultaneously determine the occupancy status for known species of concern and also provide new information about the existence of previously unknown genetic lineages on the landscape (e.g., Clusa et al., 2016). This use of eDNA sampling is particularly relevant for taxonomic groups of conservation concern for which current taxonomies are incomplete and morphological identification is difficult.

One example of a cryptic and taxonomically incomplete group of conservation concern are the freshwater snails of North America. Freshwater snails have limited dispersal abilities, narrow environmental niches, and often persist in geographically isolated habitats—all of which contribute to high levels of endemism and cryptic speciation (Hershler, Liu, & Howard, 2014; Johnson et al., 2013; Stevens et al., 2022; Young et al., 2021). This group is a focus of conservation due to pervasive habitat degradation of their native waters and competition with nonnative species invasions (Hershler, Liu, & Howard, 2014; Johnson et al., 2013). The conservation status of the most species‐rich family of freshwater snails, Hydrobiidae, is of particular concern. Of the 185 described Hydrobiidae species in North America, 16 genera and 170 species are regarded as imperiled and 14 species are listed under the U.S. Endangered Species Act (Johnson et al., 2013). Most taxa (*n* = 139) within this family are in the genus *Pyrgulopsis*. They are primarily found in the southwestern United States and often inhabit isolated perennial springs, from which they derive their common name, springsnails (Hershler, Liu, & Howard, 2014). This genus is of even greater concern because it is known to contain cryptic taxa that have yet to be described and additional lineages that have yet to be discovered (Hershler & Liu, 2017; Hershler, Liu, & Howard, 2014; Hershler, Ratcliffe, et al., 2014; Liu et al., 2017).

Environmental DNA sampling is a powerful tool for detecting freshwater snails—and *Pyrgulopsis* springsnails in particular—for several reasons. First, across much of their range, occupancy status in potential habitats has yet to be determined or updated for *Pyrgulopsis* (Hershler, Liu, & Howard, 2014). Thus, rapid eDNA sampling protocols will allow large landscapes to be comprehensively and frequently inventoried by crews with limited training. Second, morphological identification of *Pyrgulopsis* taxa is difficult, such that most current sampling for this group is reliant on destructive genetic analyses (Liu et al., 2003; McKelvey et al., 2020). This is both expensive and potentially problematic as some species are federally protected (e.g., Proposed Rule 80 Federal Register 2015‐25058). Similarly, eDNA sampling creates the potential to sample haplotypes from across an entire population of springsnails (or at least more than the 10–30 individuals typically collected for destructive analysis), which enables greater power to detect low‐frequency haplotypes. In the case of newly discovered species, conventional sampling will still be required to perform morphological descriptions for species designation, but these sites likely represent a minority of cases.

The high levels of genetic diversity among *Pyrgulopsis* mitochondrial haplotypes (Hurt, 2004; Lavretsky et al., 2021; Liu et al., 2017) also presents some possible challenges for taxon‐specific eDNA sampling. Although taxon‐specific qPCR is a sensitive and cost‐effective tool for species detection, the high levels of endemism and the prevalence of many undescribed species within this group (Hershler, 1998; Hershler, Liu, & Howard, 2014) preclude a taxon‐specific approach. Instead, we must rely on metabarcoding/amplicon sequencing‐based approaches that allow for a broader taxonomic scope. Executing this effectively requires careful attention to primer development because current amplicon sequencing protocols (i.e., metabarcoding) can be highly sensitive to amplification bias (Kelly et al., 2019).

In this study, we use the genus *Pyrgulopsis* as a case study to develop and demonstrate a pipeline for species detection and discovery based on eDNA metabarcoding. We build group‐specific primers by assembling a cytochrome oxidase subunit I (COI) sequence alignment and phylogenetic tree for *Pyrgulopsis* taxa in North America and target a taxonomically informative portion of the gene. We then collected eDNA samples from across the southwestern United States and compared the effectiveness of this approach for population detection with conventional sampling as part of a multi‐year monitoring program in a portion of the study area.

## 2 METHODS

### 2.1 Primer and positive control development

To develop degenerate primers to preferentially amplify *Pyrgulopsis* species, we first ensured that we had representative sequences spanning the breadth of *Pyrgulopsis* diversity. To accomplish this, we constructed a phylogenetic tree for *Pyrgulopsis* using COI sequences available from GenBank as of June 2023. After filtering the dataset to exclude multiple representatives from the same species complex, we constructed an alignment of 137 taxa (131 *Pyrgulopsis*, six outgroup taxa) using MAFFT v7.215 (Katoh & Standley, 2013), including the majority of described *Pyrgulopsis* species. The resulting 658 base pair (bp) alignment was partitioned by codon position into three ~220 bp character sets. We fit nucleotide substitution models to each character set using ModelFinder (set to merge partitions for a better fit) as implemented in IQTREE 2.1.3 followed by maximum likelihood tree inference with 1000 ultra‐fast bootstrap replicates (Chernomor et al., 2016; Hoang et al., 2018; Kalyaanamoorthy et al., 2017; Lanfear et al., 2012; Minh et al., 2020). Limited phylogenetic signal for resolving deeper nodes within the group led us to perform 15 independent searches in order to ensure convergence of likelihood values among multiple runs (command line: iqtree2 ‐s *Alignment_file.fa* ‐p *partition_file.nex* ‐runs 15 ‐m MFP + MERGE ‐B 1000 ‐nt 2). The resulting maximum likelihood topology revealed a monophyletic *Pyrgulopsis*, with four weakly supported primary clades. Since the included *Pyrgulopsis* species formed a monophyletic clade in our phylogenetic analysis we elected to use this taxon set for downstream analyses and primer construction (The alignment file, codon partition file, and resulting tree are available on Dryad https://doi.org/10.5061/dryad.tb2rbp07q, Vanderpool et al., 2023a).

We designed degenerate primers capable of amplifying the 3′ region of the *Pyrgulopsis* mitochondrial gene COI. Similar to other universal COI primers used in metabarcoding (Elbrecht & Leese, 2017; Folmer et al., 1994; Geller et al., 2013), the reverse primer was designed to exploit a conserved region near the 3′ end of the gene. To increase specificity for *Pyrgulopsis* we reduced the degeneracy from that of the BR2 primer described by Elbrecht and Leese (2017); 144‐fold degenerate versus 196‐fold degenerate in the original primer. However, since all but one of the 131 *Pyrgulopsis* COI sequences obtained from GenBank were amplified and sequenced using this same reverse priming site, the priming site sequence was not available for alignment. Instead, we performed BLAST searches with *Pyrgulopsis* COI queries to identify the most similar gastropod species where the priming site sequence was available. We then aligned the gastropod COI sequences identified with this search to our *Pyrgulopsis* representatives and used the states present in these taxa to reduce the degeneracy in the reverse primer. The forward primer is a version of BF1 described by Elbrecht and Leese (2017), modified to decrease degeneracy (96‐fold degenerate versus 128‐fold degenerate) according to our alignment of 131 *Pyrgulopsis* COI sequences described above.

The true number of *Pyrgulopsis* species is unknown, making it difficult to ascertain whether our primers with decreased degeneracy may fail to detect some new species (Eloe‐Fadrosh et al., 2016). The forward priming site is invariant at the first and second codon positions; however, all six of the third codon positions are 2‐ to 3‐fold degenerate in our alignment. To evaluate the potential for failing to detect undescribed *Pyrgulopsis* taxa, we constructed a test dataset containing hypothetical sequences with 1–3 primer‐template base pair mismatches in the forward primer, reverse primer, or both. We then conducted an in silico test of the effects of potential mismatches on our ability to amplify *Pyrgulopsis* DNA using *eDNAssay* (Kronenberger et al., 2022), a random forest classifier, to predict amplification of these hypothetical templates with up to three base pair mismatches at each primer site.

The primers described above were tested for effectiveness using pure extractions of DNA from three species: *P. ignota*, *P. metcalfi*, *P. texana*. Because these amplifications were successful (data not shown), we tested these primers using known positive eDNA extracts (based on *16S* sequencing), as well as extracts thought to be negative for *Pyrgulopsis* DNA to gauge the effectiveness of amplifying target species in a mixed extract. We added an Illumina adapter sequence to our primers for the second indexing‐PCR step that includes a unique barcode identifier for each sample.

Pyrg_BF1_tailed‐GTGACTGGAGTTCAGACGTGTGCTCTTCCGATCT **ACBGGRTGRACYGTRTAYCC**

Pyrg_BR2_tailed‐ACACTCTTTCCCTACACGACGCTCTTCCGATCT **TCDGGRTGHCCRAARAAYCA**

It is common for eDNA extractions to contain PCR inhibitors, motivating the addition of an inhibitor removal step to our protocol (McKee et al., 2015). However, even with this additional step, the possibility of residual inhibitors can result in a false negative test. In order to detect possible inhibition, we developed a positive PCR control using synthetic mitochondrial sequence from the extinct thylacine (Tasmanian Tiger, *Thylacinus cynocephalus*). Priming sites consisting of *Pyrgulopsis* sequences compatible with our degenerate primers were added to the end of the thylacine sequence, resulting in an amplification product 100 bp shorter than the amplicon created from true *Pyrgulopsis* templates. The use of thylacine DNA makes it possible to distinguish potential contamination from the positive control from that of background contamination derived from extant species present in the lab space (very few molecular laboratories are actively working on museum specimens of thylacines). The shorter amplicon size makes it possible to spike a small amount of positive control into one of the sample PCR replicates to test for reaction inhibition but differentiate the thylacine control from *Pyrgulopsis* targets on an agarose gel. We assumed there was PCR inhibition if all the reactions, including those with the positive control, did not amplify. If only the reaction containing the positive control amplified, and the product was 100 bp shorter than expected, we assumed the sample was not inhibited, but that it was negative for target species DNA. When all or some of the replicates amplified, the one containing the positive control had a double band, was free from inhibitors, and potentially contained amplicons for the target species. We then selected potentially positive samples for indexing PCR and sequencing.

For this project, the linear, synthetic gene for the thylacine was purchased from Integrated DNA Technologies (gBlock; IDT), re‐suspended in Tris‐EDTA, and quantified on a Qubit fluorometer (ThermoFisher Scientific) prior to dilution to the target copy number concentration. The positive control sequence with priming sites underlined is: 5′‐ACTGGGTGGACCGTGTATCCTCATCACA ACAATTATCAATATAAAACCTCCAGCCCTATCCCAATATCAAACTCCATTATTTGTTTGATCAGTAATAATTACAGCAGTACTTCTACTATTATCCTTACCCGTTCTGGCAGCAGGCATTACAATACTACTTACGGACCGAAATCTTAATACAACATTTTTTGACCCTGCTGGAGGAGGTGACCCAATCCTTTATCAACATCTATTTTGATTTTTTGGTCACCCTGA‐3′.

### 2.2 Sampling and analysis

Our goal was to characterize springsnail biodiversity focusing on habitats with few or no historical data on these taxa. We conducted eDNA sampling in potential springsnail habitats, based on previous spring surveys or satellite imagery, on or near five U.S. Department of Defense installations in the southwestern U.S.: Fallon Range Training Complex (60 samples from 60 sites; Pershing and Churchill Counties, Nevada), Fort Huachuca (52 samples from 44 sites; Santa Cruz County, Arizona; including locations on Coronado National Forest and The Nature Conservancy Ramsey Canyon Reserve), Camp Navajo (4 samples from 4 sites; Coconino County, Arizona), Nellis Air Force Base (22 samples from 21 sites; Nye and Clark County, Nevada), and Camp Williams (2 samples from 2 sites; Salt Lake County, Utah). We also collected two additional samples on Bureau of Land Management lands in Utah (Figure 2). Samples were collected by U.S. Forest Service, Springs Stewardship Institute, Arizona Game and Fish Department, and Department of Defense crews. All sampling on Department of Defense lands was performed by either local installation personnel or sampling crews with research sampling permits and permissions.

We collected water samples following the protocol described by Carim, Dysthe, et al. (2016), Carim, McKelvey, et al. (2016) and Wilcox et al. (2016). Briefly, at each site we filtered up to 5 L of water through one to three 47 mm diameter, 1.5 μm pore‐size glass filters (GE Healthcare; www.gehealthcare.com) using a 12 V peristaltic pump. At each site, our goal was to filter 5 L of water. If the first filter paper clogged before reaching this goal, we continued filtering with a second filter. We continued with additional filter papers until we reached 5 L of total water or three filter papers. When necessary, multiple filter papers per site were combined during DNA extraction (below). We removed each filter with sterile forceps, folded it into quarters, and placed the filter in a sterile bag containing silica desiccant. For each filter paper collection, we used new, clean collection materials including the filter funnel and forceps. All other collection apparatus, including suction hoses and buckets, were washed with a 10% household bleach solution (approximately 0.6% sodium hypochlorite) to prevent cross contamination and inadvertent species transfer between sites. The desiccated samples were maintained at ambient temperatures for no more than 2 weeks before being transferred to a −20°C freezer prior to DNA extraction.

Extractions were performed in a dedicated eDNA extraction room kept free of PCR products and other potential background contamination in accordance with the National Genomics Center for Wildlife and Fish Conservation eDNA Program Standard Operating Procedure. We cut each filter in half; one half of the filter was archived at −20°C for future analyses and the other half was extracted using a modified DNeasy Blood and Tissue Kit (QIAGEN) protocol. We followed the manufacturer's protocol for extraction from animal tissue with the modifications described in Carim, Dysthe, et al. (2016), Carim, McKelvey, et al. (2016). In cases where a sample consisted of multiple filters, we extracted each filter separately and then spun them through the same silica spin column (see Carim, Dysthe, et al., 2016). Extractions were eluted in 100 μL of Tris‐EDTA and then passed through a OneStep™ PCR Inhibitor Removal Kit column (Zymo Research) and stored at −20 or −80°C until PCR amplification.

PCR reactions were composed of 500 nM each primer (forward and reverse), 2 mM MgCl_2_, 2 ng BSA, 12.5 μL KAPA HiFi 2× HotStart ReadyMix (Roche, Inc.), 4 μL template, and molecular grade water to a total reaction volume of 25 μL. Reactions were run in quadruplicate with 1 μL of the positive control added to one reaction in order to test for PCR inhibition. Thermal cycling parameters were designed to favor primer‐template exact matches using a touchdown protocol. Samples were subjected to an initial denaturation at 95°C for 3 min, followed by 98°C for 20 s and then annealing temperatures starting at 67°C for 60 s, followed by a 20 s extension step at 72°C. The annealing temperature was decreased by one degree each cycle for 10 cycles until the optimal annealing temperature of 57°C was reached. This touchdown phase was followed by 40 cycles at 57°C with a 3‐min extension step on the final cycle. The resulting PCR products were visualized on a 1% agarose gel, with successful reactions combined, cleaned using 0.9× Ampure XP beads (Beckman Coulter), and eluted in 20 μL of 10 mM Tris‐EDTA. Illumina sequencing adaptors and barcodes were then added to each cleaned amplicon reaction via a 15 cycle indexing PCR consisting of the following reaction components: 3 μL cleaned amplicon PCR product, 500 nM Illumina P5 adaptor with unique barcode, 500 nM Illumina P7 adaptor with unique barcode, 7 μL H_2_O, and 12.5 μL KAPA HiFi 2× HotStart ReadyMix (Roche, Inc.) to a final reaction volume of 25 μL. Cycling parameters consisted of an initial denaturation of 98°C for 30 s, followed by 98°C for 10 s, 60°C for 20 s, and 72°C for 20 s. Indexed PCR products were cleaned using 0.9× Ampure XP beads and eluted in 20 μL of 10 mM Tris‐EDTA. The concentration of each cleaned product was estimated using a Qubit fluorometer. Thirty to forty indexed samples were then pooled in equimolar ratios (~80 ng of each indexed sample) with the final volume cleaned using 0.9× Ampure XP beads. This third cleaning step is critical for the complete removal of primer dimers generated during the preceding PCR steps. The concentration of the cleaned, pooled library was estimated using both a Qubit fluorometer and an Agilent 2100 Tapestation.

Pooled libraries were sequenced using a 2 × 250 cycle Illumina MiSeq Nano kit on four separate sequencing runs. However, one of these runs resulted in low yield and was sequenced a second time. For the second sequencing attempt, we selected seven sites and prepared two new indexed libraries from the same extractions as the first sequencing attempt. We sequenced these two libraries independently to verify the results of the initial low‐yield sequencing run. Finally, we prepared two libraries for two of our samples for which we knew *Pyrgulopsis* were present in order to evaluate reproducibility in the detected haplotypes.

We processed reads using a pipeline based on the DADA2 package (Callahan et al., 2016) in *R* (R Core Team, 2020). We used the DADA2 function filterAndTrim to remove sequences with ambiguous bases or more than two expected errors based on quality score estimates, to trim off primer sequences, truncate sequences at the first quality score of four or to a maximum length of 250 bp, and remove any sequences <25 bp after trimming. We then used DADA2 to estimate error rates from our sequences using pseudo‐pooled sampling, inferred amplicon sequence variants (ASVs), merged paired‐end reads, and removed chimeric sequences. Next, we performed a BLAST search (Camacho et al., 2009) with *Pyrgulopsis marcida* COI sequence as a query to identify gastropod sequences close to *Pyrgulopsis* that were retained after filtering (command line: blastn ‐query ../Pyrg_marcida_COI.fa ‐subject All_MiSeq_combined_DADA2_filtered.fa ‐evalue 1e‐50 ‐outfmt 6). The resulting sequences were subjected to further downstream inspection to eliminate any remaining errors. Three low‐abundance ASVs were divergent from more common haplotypes found at the same site (typically <1% divergent from one another). The three ASVs differed in the ~60 bp near the 5′ or 3′ end but were otherwise identical to the common haplotype. A BLAST search using this divergent region suggested that these were chimeric sequences, as the closest hit in each case was a species common in freshwater environments. Consequently, we inspected the remaining ASVs approximately 5% divergent from a common ASV and discovered another chimeric sequence with moderate abundance (322 reads) which was removed from downstream analyses. Additionally, in two instances a haplotype identified as unique was the reverse complement of the region of interest. These sequences were collapsed into their respective haplotypes. To identify potential contaminants, we explored detections with at or near 100% matches to a known sequence, but where the known distribution of the species of interest was geographically incongruent with the site in which the detection was made. Additionally, we used BLAST to compare *Pyrgulopsis* ASVs identified in our sequencing effort to a comprehensive FASTA file of springsnails previously sequenced for COI at the National Genomics Center for Wildlife and Fish Conservation (*n* = 2955).

**FIGURE 1:**
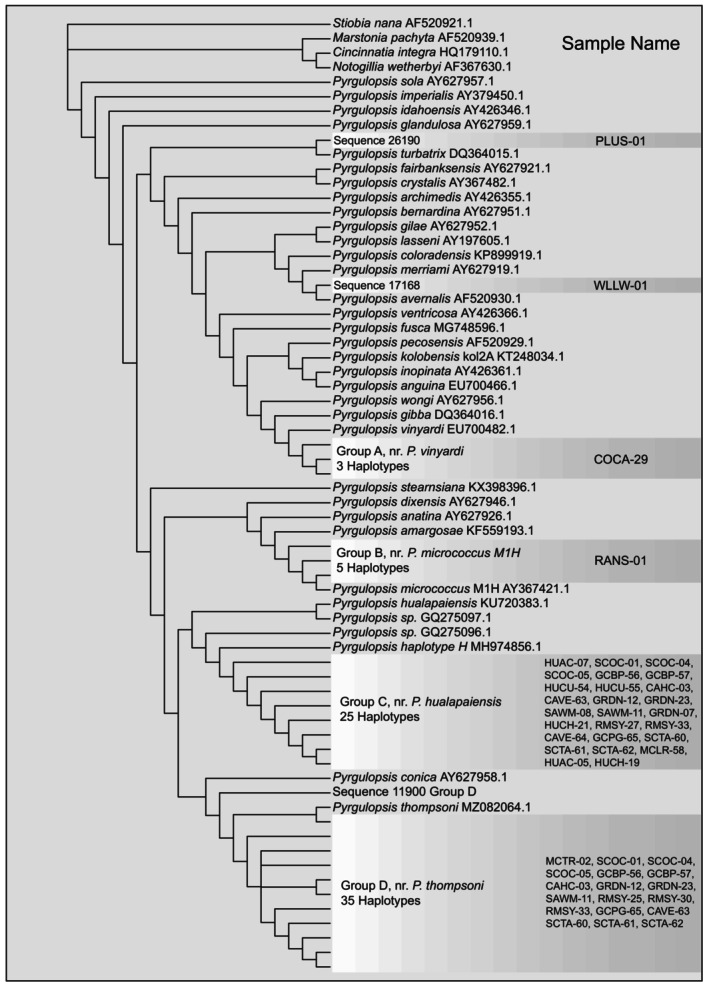
The maximum likelihood phylogeny estimated from 137 COI sequences obtained from GenBank and the 70 ASVs recovered in this study. Taxa were pared from the tree for visualization purposes. Most of the ASVs cluster into four clades (Groups A–D), identified by the nearest named species included in the phylogeny. Where multiple haplotypes were not identified for a particular ASV the sequence ID is given in bold (*P. avernalis* and *P. turbatrix*).

To assess the taxonomic affiliation of the ASVs retained after filtering, we added them to our original alignment, then estimated a phylogenetic tree using IQ‐Tree 2.1.3 with 15 independent runs using the *‐‐runs* flag implemented in IQ‐Tree and retained the topology with the highest likelihood (command line: iqtree2 ‐s Alignment_file.fa ‐p partition_file.nex ‐‐prefix Name_of_run ‐‐runs 15 ‐o Outgroup_name ‐m MFP ‐B 1000 ‐nt 2). The majority of taxa were then trimmed from the resulting 207 taxon tree for visualization purposes (Figure 1).

### 2.3 Comparison with conventional sampling

We compared results of our eDNA sampling and analysis to data from conventional sampling at 18 sites to determine concordance between *Pyrgulopsis* detection methods. We collected 1–5 eDNA samples in either 2019 or 2020 from these 18 sites. Conventional samples were collected by a long‐term *Pyrgulopsis* monitoring program in the Fort Huachuca area (Tullo & Sorensen, 2021). This program sampled the 18 sites for *Pyrgulopsis* detection 1–4 times from 2016 to 2020. Conventional surveys were conducted using active, timed visual searches of springsnails on natural substrates, typically lasting 10 to 20 min or until first springsnail detection. All observed *Pyrgulopsis* were considered to be Huachuca springsnail and did not undergo further morphological or molecular analysis. Additionally, conventional sampling detections were sometimes variable over time and it is unclear to what extent this variation is due to imperfect detection versus year‐to‐year extirpations and recolonizations of local *Pyrgulopsis* populations. To attempt to control for this, we considered any site with at least one detection in 5 years to be a positive detection for our methods comparison.

## 3 RESULTS

In addition to testing primer performance on DNA from several *Pyrgulopsis* taxa, we evaluated primer generality against potential, unseen diversity in *Pyrgulopsis* using an in silico tool (*eDNAssay*; Kronenberger et al., 2022) and hypothetical templates with introduced mismatches (see Section 2). Even with up to four mismatches (two per primer), assignment probabilities were ⋝0.76. With up to three mismatches in a single primer, eDNAssay assignment probabilities were ⋝0.81. Kronenberger et al. (2022) found that 100% of primer sets with assignment probabilities >0.7 amplified. This is an unrealistically large number of mismatches as the first and second codon positions located in the priming sites are fixed across the *Pyrgulopsis* examined here as well as highly conserved among divergent outgroups. The generality of these primers was further supported by the recovery of ASVs from divergent gastropods from environmental samples for which the nearest BLAST hits were to genera outside of Hydrobiidae, (e.g., *Turbo*, *Valvata*, *Glyptophysa*, *Rabdotus*, *Biomphalaria*, *Ilanga*, and *Muticaria*). None of the divergent ASVs recovered were close matches to these genera, likely meaning they were derived from species not represented in GenBank. Across these in silico and in vitro tests, we found strong evidence that the primer sets and PCR conditions used in this study were sufficient to sample all previously described *Pyrgulopsis* taxa and the range of variability that could be reasonably expected in as‐of‐yet undescribed taxa from this group.

### 3.1 Sequencing and quality control

Our sequencing effort resulted in 53,894,920 paired‐end reads (Vanderpool et al., 2023b). After filtering, denoising, and removing chimeric sequences using the DADA2 pipeline, these were pared to 30,280,265 reads across 56,171 ASVs. The majority of these reads were not *Pyrgulopsis* in origin (97.71%). Using BLAST to identify *Pyrgulopsis* haplotypes resulted in 127 ASVs, from which four chimeric sequences and two reverse complemented sequences were excluded from downstream analyses. Inspection of the remaining 121 ASVs revealed a subset of 15 detections with low read abundance (<50 reads) that were subjected to further inspection.

Among these low read abundance ASVs, there were two cases where a distinct ASV was detected at only a single site. The first of these was a close match to *P. avernalis*, the Moapa Pebblesnail (99.65% identity, 39 reads, sequence I.D. 17168, Table S1). This sequence was found at Plummer Springs (PLUS‐01) on the Moapa Valley National Wildlife Refuge and was retained as a true positive as it is known from this area (Hershler, 1998; Hershler & Liu, 2017; Ledbetter et al., 2023). The other ASV with low abundance (15 reads, sequence I.D. 26190, Table S1) was retained as a true positive because it had 100% sequence identity with *P. turbatrix*, the Southwest Nevada Pyrg, and was collected from Willow Springs (WLLW‐01), a site from which the species had been previously collected (Hershler, 1998; Ledbetter et al., 2023). For the remaining *Pyrgulopsis* ASVs with abundance <50 reads, detected at only a single site, the ASV differed by only 1–2 base pairs from a more common haplotype or could be traced to a background contamination event (see below). We conservatively elected to remove these ASVs (*n* = 13) as potential false positives.

Our BLAST search of 2955 COI sequences previously processed in the lab identified nine sites (six species, 38 ASVs) where an ASV shared 100% identity between sites that were separated by >500 miles. Further inspection revealed these sites were processed in the same batch of amplifications, leading us to suspect these results were due to background contamination in a common PCR reagent. We elected to remove detections at these sites for the ASVs incongruent with known species distributions.

The remaining 70 ASVs primarily clustered into four clades (arbitrarily designated here as Groups A–D): Group A, the nr.‐*P. vinyardi* clade (*n* = 3 ASVs); Group B, the nr.‐*P. micrococcus* M1H clade (*n* = 5 ASVs); Group C, the nr.‐*P. hualapaiensis* clade (*n* = 25 ASVs); and Group D, the nr.‐*P. thompsoni* clade (*n* = 35 ASVs). These groups, further described using the names of their sister taxon in Figure 1, clustered together with each group containing ASVs that differed from another ASV or the sister taxon at only a few nucleotide sites (usually 1–2). In order to aid in visualization, these groups are collapsed in size in Figure 1, and represented by blocks labeled with locality information. Group A consists of three haplotypes found at Coyote Canyon Spring (39.545038, −118.225527) a small drainage on the edge of the Dixie Valley in central Nevada. These haplotypes are closely related to *P. vinyardi* (99.66%, isolate: SM38A, GenBank: EU700482) and *P. gibba* (99.66%, isolate: P134D, GenBank: AY426359) where *P. gibba* has been previously collected in Churchill county, near Dixie Valley (Springsnail Conservation Team, 2020), but to our knowledge this is the first detection of this species at this locality.

**FIGURE 2:**
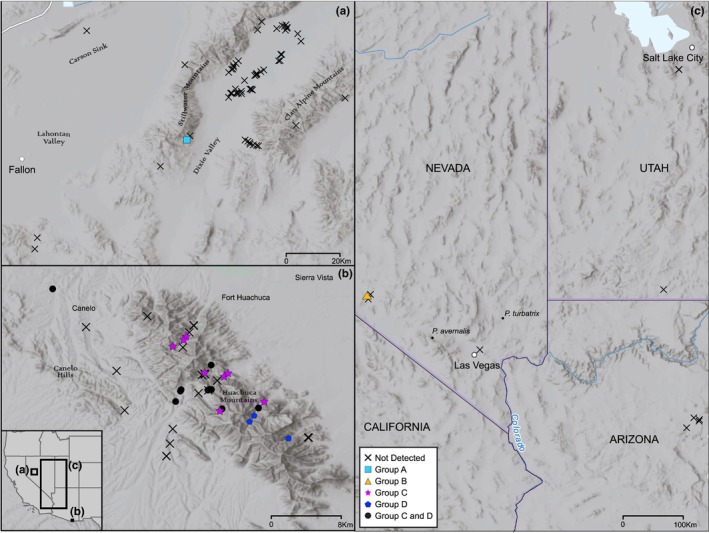
eDNA survey sites included in this study. Filled shapes represent sites where *Pyrgulopsis* was detected while “X” represents no detection. Panel ‘a’ depicts sampled sites around Fallon, NV area. Panel ‘b’ depicts sites sampled in the Huachuca, AZ region. Panel ‘c’ depicts sites across the Great Basin, in Utah, Nevada, and Arizona. Service Layer Credits: ESRI, HERE, Garmin, (c) OpenStreetMap contributors, and the GIS user community.

Group B is comprised of five sequences found at site 7 J Ranch‐9 Spring (RANS‐01) identical or nearly identical to *P. micrococcus* M1H. 7 J Ranch‐9 Spring is located in southwestern Nevada (37.03795, −116.7123) near the area where *P. micrococcus* M1H was first discovered (Liu et al., 2003) (Figure 2). Group C haplotypes, first identified by Hurt (2004), were detected at 26 sites (See Table S1) all within the Huachuca region (See Figure 2 map). Many of these sites overlap with previous detections of this lineage from the same area (Hershler et al., 2016; Hurt, 2004). It is a similar case for Group D, comprised of 35 ASVs identical to or nearly identical to *P. thompsoni*, the Huachuca Springsnail. These 35 ASVs are distributed among 18 different sites throughout the Huachuca region, many of which overlap with sites where Group C also occurs (See Figure 2 and Table S2 for details). *Pyrgulopsis thompsoni* is described from this region (Hershler & Landye, 1988) and has been previously sampled from sites included in this study.

### 3.2 Comparison with conventional sampling

Conventional sampling and eDNA sampling concurred on the presence of *Pyrgulopsis* at all but one site. *Pyrgulopsis* were not detected by either method at five sites. At 12 sites in which conventional sampling detected *Pyrgulopsis*, 24 of the 26 eDNA samples were positive (92.3% detection rate), including at least one replicate at every site (Table S3). At one site, eDNA sampling detected *Pyrgulopsis* where none had been collected with conventional sampling. This site, however, was in the same basin and <2 km from a site with a previous conventional detection. Because this site, SC02 was weakly positive for *Pyrgulopsis* eDNA (four reads) and only 50 m downstream from site SC01, which was a strongly positive eDNA site; this detection may represent a degraded eDNA signal from the upstream population.

## 4 DISCUSSION

Here we performed the first, to our knowledge, application of eDNA sampling for *Pyrgulopsis* springsnails. Environmental DNA sampling and sequencing detected springsnails of the genus *Pyrgulopsis* (and likely closely related genera) with results highly concordant with conventional sampling strategies. Among the 142 sites sampled here, 34 resulted in detections (24%), with the majority of these detections concordant with historical understandings of species distributions. For example, where springsnails were detected in the most recent netting‐based survey in the Huachuca landscape, 94.4% were also positive with eDNA sampling. Additional detections with eDNA sampling on this landscape were consistent with eDNA transport from a nearby, hydrologically connected site with known historical species occurrence. Further, the performance of *Pyrgulopsis* eDNA sampling here is consistent with the literature on morphologically similar invasive hydrobiid New Zealand Mudsnails (*Potamopyrgus antipodarum*) (Goldberg et al., 2013; Ponce et al., 2021; Woodell et al., 2021). Multiple studies have found that eDNA sampling for *P. antipodarum* can be effective, even for low density population detection. For example, Goldberg et al. (2013), using a taxon‐specific qPCR assay, were successful in detection of *P. antipodarum* at densities as low as one individual per 1.5 L of water. In natural systems, Ponce et al. (2021) reported concordance in eDNA sampling and conventional kick‐net sampling for *P. antipodarum* across six streams in Washington (USA) and Woodell et al. (2021) reported detection of a new invasion with eDNA sampling in Pennsylvania (USA), which was later confirmed with conventional sampling.

Our detection results for low‐density populations might be further improved by optimizing the field sampling design. Particularly in complex habitats, detection can be limited by sampling proximity to patchily distributed taxa because of limited spatial dispersion of the eDNA signal (Bedwell & Goldberg, 2020; Brandão‐Dias et al., 2023; Shogren et al., 2017; Wilcox et al., 2015). When the location of optimal habitat for the focal taxon is unknown, detection might be accomplished by repeated sampling over space, but when the preferred habitat is known, then sampling might target those locations (Bedwell & Goldberg, 2020). *Pyrgulopsis* are often closely associated with spring heads, so while subsampling over space may be effective (Bedwell & Goldberg, 2020), it may also be effective to simply target spring heads in complex habitats or springs with large pool habitats. An additional consideration is sample timing relative to spring hydroperiod. Sampling for eDNA requires sufficient surface water, but sampling during wet periods could also result in wasted effort on ephemeral waters that do not represent suitable springsnail habitat.

When sampling was scaled up to encompass entire landscapes, we observed that good detection rates at the local scale, translated into landscape patterns of detection that were largely concordant with previous conventional sampling. In the Huachuca region, we detected Ptho5 (and associated haplotypes Group C, Figure 1) as well as *P. thompsoni* (and associated haplotypes Group D, Figure 1), both taxa previously collected from springs in this area (Hershler & Landye, 1988; Hurt, 2004). Additionally, our comparisons with sites for which conventional sampling data was available were 94.4% concordant (17/18 sites) with the only discrepancy coming from a weakly positive eDNA detection (four reads) at a location where springsnails had not been previously reported. High concordance (>90%) among sites that are known to host springsnails and sites that do not is an indicator that the assay is performing as intended. In the Fallon region, we detected snails from the *P. vinyardi/gibba* species complex. While we are not aware of a previous detection from this exact site, this finding is unsurprising in that *P. gibba* was previously collected at many sites in the area and is common throughout central Nevada and into California (Springsnail Conservation Team, 2020). The three remaining species detected were from other parts of southern Nevada outside of the Nellis AFB region. *Pyrgulopsis micrococcus* isolate M1H and associated haplotypes (Group B, Figure 1) were isolated from a spring (RANS‐01) in a region where multiple *P. micrococcus* lineages have been identified (Liu et al., 2003). The remaining two detections were of low abundance (<50 reads) but from springs where these species have been previously reported. *Pyrgulopsis avernalis*, the Moapa Valley Springsnail, was detected in Plummer Spring (PLUS‐01) in the Moapa Valley National Wildlife Refuge, the area for which the species is named. The final low abundance detection was of *P. turbatrix* from Willow Springs (WLLW‐01), a site from which this species has been previously collected via conventional sampling and was thus unsurprising. While these ASVs with low abundance reads are likely true positives, these detections should be verified with additional sampling.

The uncertainty associated with some detections in this study is not unique. Environmental DNA metabarcoding for rare species currently faces a fundamental signal‐to‐noise problem: Both true presence of rare species and low‐level contamination tend to result in low read abundances. Applying thresholds that discard reads below some threshold abundance is a common approach to remove contamination (Drake et al., 2022), but this practice can also result in the loss of true detections and impact ecological inference (Littleford‐Colquhoun et al., 2022; Young et al., 2013). In this study, suspected contamination events tended to have low read abundances (mean = 892 range = 1–17,812 reads/library in those discarded), but this overlapped putative real detections (mean = 9133, range = 15–326,382 reads/library), including several with very low read counts that were almost certainly accurate. For example, we detected just 39 reads for a haplotype very similar to a reference for *P. avernalis*, the Moapa Pebblesnail (99.65% identity) at Plummer Springs on the Moapa Valley National Wildlife Refuge. This was almost certainly a true positive detection because (1) this taxon was never otherwise handled in the lab or detected in our study and (2) is known to occupy this area (Hershler, 1998; Hershler & Liu, 2017; Ledbetter et al., 2023). With current metabarcoding technologies and risks of contamination during both library preparation and sequencing (i.e., tag switching and run‐to‐run carry‐over), distinguishing true positive detections and contamination based on read abundance alone may not be possible. In this study, we took a conservative approach to inference of species presence by discarding low‐abundance detections unless there was outside information suggesting species presence and discarding any other detections associated with a suspected library preparation contamination event. However, the ultimate solution for ambiguous or uncertain detections is likely to be repeat sampling. Persistent populations should result in persistent eDNA signals over time, even if those persistent signals are weak. Thus, repeated detection of low‐abundance haplotypes over repeated sampling, library preparation, and sequencing efforts provides strong evidence of true species presence. Environmental DNA sampling may also play a complementary role to conventional sampling where rapid eDNA sampling can identify habitats for re‐sampling with conventional approaches which could establish species presence with a higher level of certainty. Before making conservation management decisions based on specific detection data from this study, we recommend repeated sampling with eDNA or conventional approaches to confirm new detections.

Another uncertainty that is general to current eDNA metabarcoding tools concerns real versus error‐derived haplotype diversity. A unique attribute of eDNA sampling is that the DNA of a potentially large number of individuals are present in a sample. In our system, our water samples could potentially contain DNA from hundreds of individuals, while most conventional sampling typically results in sequencing of just 1–30 individuals from a site (Liu et al., 2003; McKelvey et al., 2020). Thus, there is potential to detect greater haplotype diversity, including rare haplotypes. Simultaneously, it is well known that PCR and high‐throughput sequencing generate errors which can be difficult to distinguish from real biological diversity. Historically, operational taxonomic unit (OTU) approaches have compressed both real biological and sequencing error‐derived diversity by representing a range of haplotypes with centroid or representative sequences. In microbial systems, this approach resulted in the loss of meaningful biological inference at fine scales, motivating the development of denoising algorithms which attempt to correct for sequencing error to estimate original template sequences. However, even the best performing algorithms generate some spurious reads (i.e., inference of haplotypes with sequencing errors; Callahan et al., 2016). The diversity of haplotypes detected here likely represent a composite of both real biological diversity detected due to deep sampling of individuals from environmental samples and sequencing errors. In cases where a species was particularly abundant, we observe a primary haplotype that accounts for the majority of the total reads recovered (~85%–95%), with one or two minor haplotypes accounting for most of the remaining reads followed by many rare haplotypes of low abundance (see Table S1, Groups C and D). These rare haplotypes often differ by only 1–2 base pairs from the primary haplotype with the mutation often being a non‐synonymous change. Since the majority of differences we observed in our alignment of 131 *Pyrgulopsis* species are synonymous substitutions, it stands to reason that these rare haplotypes with non‐synonymous changes could represent sequencing errors. However, these rare haplotypes may also represent somatic mutations within large populations that are not typically observed using Sanger sequencing.

With an appreciation of the potential limitations, this study suggests several ways in which eDNA sampling can be a useful tool in the toolbox for research and monitoring of *Pyrgulopsis* and other cryptic, species‐rich, and incompletely described taxa. Compared to conventional sampling, eDNA sampling is more time efficient, requires minimal personnel training, and is likely more cost effective for species detection. We have found that trained field technicians can struggle to distinguish even deeply divergent mollusk taxa (e.g., *Pyrgulopsis* versus immature *Physa*); >400 MYA divergence (McKelvey et al., 2020). For these cryptic taxa, molecular analyses are still required. A typical conventional sampling approach for *Pyrgulopsis* may require a field crew to net 10–30 individuals, then submit these for barcoding. At a cost of $30/barcode, this translates into a cost of at least $300/site. Using a MiSeq platform, we estimate our cost/sample in this study to be approximately $150. With increased sample multiplexing, samples could be sequenced on a higher capacity platform, further reducing the per sample cost. Furthermore, existing eDNA sampling protocols (e.g., Carim, Dysthe, et al., 2016; Carim, McKelvey, et al., 2016) can be incorporated into conventional springsnail surveys or piggy‐backed with non‐springsnail surveys in remote areas, reducing the logistical cost of sampling.

With the increasing use of eDNA methods over the last two decades has come an increasingly complete suite of tools and publications addressing eDNA sampling best practices, project planning, and application (e.g., Goldberg et al., 2016; Kelly et al., 2023; Liu et al., 2020; Maloy et al., 2023) to help guide potential users seeking to partner with molecular labs.

This study demonstrates that partnerships between existing springs inventory and molecular laboratories can deploy eDNA sampling as a rapid, low‐cost way to identify putative occupied sites (and rare species or haplotypes) for further examination. Just as conventional barcoding has revolutionized our view of cryptic biodiversity at global scales (Hajibabaei et al., 2007), we anticipate that eDNA sampling will play an increasingly important role in characterizing unseen diversity at landscape scales.

## AUTHOR CONTRIBUTIONS

**Daniel D. Vanderpool:** Conceptualization (equal); data curation (lead); formal analysis (lead); investigation (lead); methodology (equal); supervision (supporting); writing – original draft (lead); writing – review and editing (equal). **Taylor M. Wilcox:** Conceptualization (equal); formal analysis (supporting); funding acquisition (lead); investigation (equal); methodology (equal); project administration (lead); supervision (lead); writing – original draft (equal); writing – review and editing (equal). **Michael K. Young:** Methodology (supporting); writing – review and editing (equal). **Kristine L. Pilgrim:** Data curation (supporting); methodology (supporting); resources (supporting); supervision (supporting); validation (supporting). **Michael K. Schwartz:** Funding acquisition (supporting); project administration (supporting); resources (supporting); supervision (supporting); writing – review and editing (supporting).

## CONFLICT OF INTEREST STATEMENT

The authors declare no conflict of interest.

## Supporting information


Tables S1–S4.

## Data Availability

All FASTQ files associated with this work are archived under SRA BioProject ID: PRJNA1020154. Supplementary Material is available from Dryad: https://doi.org/10.5061/dryad.tb2rbp07q.
